# Validation of Self-Reported Measures in Health Disparities Research

**DOI:** 10.4172/2155-6180.1000e114

**Published:** 2012-10-12

**Authors:** Bertha Hidalgo, Melody Goodman

**Affiliations:** 1 Department of Biostatistics, Section on Statistical Genetics, University of Alabama at Birmingham, 1665 University Blvd, RPHB 443, Birmingham, AL 35294; 2 Division of Public Health Sciences, Department of Surgery, Washington University School of Medicine, Campus Box 8100, 660 S. Euclid Avenue, St. Louis, Missouri 63110

## Abstract

Validation of self-reported measures can be achieved effectively and accurately when data collection involves objective measures that can be clinically validated. On the other hand, validation of self-reported social constructs, often used in health disparities research is a much harder task to achieve, particularly when the outcome is hard to quantify (e.g. racism, discrimination and segregation experience). We discuss validation and the challenges faced, when using current approaches in health disparities research.

Reducing racial/ethnic disparities in health is a major priority in the United States [1]; however, we lack the statistical infrastructure to establish benchmarks, monitor progress and track changes over time. One necessary component of the statistical infrastructure needed for health disparities research is to establish the validity and reliability of constructs and instruments across racial, ethnic and cultural groups [2]. Researchers need to continue to examine how racial/ethnic differences in risk aversion and preferences influence medical decision-making and health outcomes. In addition, examination of perceived discrimination, racial bias and segregation experience as social determinants of health disparities, remain legitimate research questions [3]. More studies are needed to determine whether these factors significantly contribute to health care disparities, and identify strategies to minimize or eliminate their effects on health. However, measurement and validation of self-reported social risk factors can be challenging, particularly in areas where constructs are difficult to define and/or quantify.

Validity refers to the degree to which results of a measurement correspond to the actual outcome [4]. Measurement of physical outcomes (e.g. weight, blood pressure, cholesterol) and social variables (e.g. discrimination, quality of life, racism) in public health research is inherent to the examination of complex health disparity problems. Validation requires a criterion standard; a diagnostic test that is regarded as definitive for a particular measure, and thereby becomes the ultimate measure for comparison. For disparities research, it is important to assess that the criterion standard does indeed do what it is intended to do, in diverse populations. In some cases, no clinical or physical criterion standards exist, requiring the use of instruments like questionnaires to establish validity. On occasion, some measures will not have a criterion standard at all; in such cases there is a need for other validation methods and the development of statistical methodology to validate such data.

Discrimination, racism, race and segregation experience are social constructs, which in health disparities research can be difficult to measure [5]. Validation of these self-reported measures is an even harder task to achieve. Some studies have used census data, surname analysis, and/or geocoding to validate self-reported racial/ethnic measures [6–8]. These studies have found that validation of self-reported data and measured/objective data continue to result in disagreement and differences in assessment [5–8].

For clinical outcomes that can be measured by physical means, establishing validity is relatively simple. Usually, the self-reported measurement is compared to some clinically accepted standard. For example, self-reported measures such as height, weight, hypertension and diabetes can be validated against measures of these outcomes obtained in a clinical examination [9, 10]. However, researchers are increasingly obtaining information about chronic illnesses and risk factors for disease from self-reported survey data, which have an obvious advantage over clinical records as they can be systematically collected for a large and representative sample of the population, without great expense. However, the validity of the resulting self-reported data depends on the ability of respondents to report accurate data, eliminate recall bias and ensure a willingness to report sensitive information. To assess the validity of self-reported clinical outcomes data, researchers have attempted to compare self-reported responses with medical or administrative records [11].

Although medical record is frequently viewed as a preferred source for individual level health data, routine quality assessment of medical record data is generally viewed as too costly. The primary purpose of the collection of such data is for the care of individuals, not research, which requires systematic collection across individuals. As such, medical records are often subject to error due to inconsistent recording of events such as reporting of physician orders [12], procedure and laboratory reports, and delays in physician reporting resulting in recall bias [13]. Luck et al. [13] found that using medical records may both, under report and over report care. Differences in time constraints, coordination and continuity of care, and incorporation of systems such as integrated medical records or electronic medical records may affect the quality of medical record data. Another advantage of self-reported survey data is the ability to provide information on experiences and perspectives, not routinely captured by the medical record. Self-reported data, however, are also subject to error due to problems with recall and social desirability bias, and/or general patient health knowledge [14, 15].

We illustrate though an example, validating self-reported pain assessment. One may compare the results with other self-reported measures (survey validation), use the judgement of an expert observer (clinical validation), and examine if results predict pain-related behavior like sweating, moaning, or requests for medication (physical validation) [16]. One may also assess if the measurements yield consistently different results for conditions in which the severity of pain is generally believed to vary (e.g. minor abrasion, dental extraction, etc.). The adaptation of this type of validation approach suggest promising future directions for the validation of self-reported health disparities measures (e.g. discrimination, racism, segregation experience), for which clinical, medical record and physical criterion standards do not exist. There is a need to develop statistical methodology to address the challenge of validating social constructs in health disparities research.
